# Aspirin: Pharmacology and Clinical Applications

**DOI:** 10.1155/2012/173124

**Published:** 2011-11-17

**Authors:** Enma V. Paez Espinosa, John P. Murad, Fadi T. Khasawneh

**Affiliations:** Department of Pharmaceutical Sciences, College of Pharmacy, Western University of Health Sciences, 309 East Second Street, Pomona, CA 91766, USA

## Abstract

Antiplatelet therapy has been documented to reduce risks of cardiovascular disease after acute myocardial infarction, coronary artery bypass graft, and in chronic atrial fibrillation patients, amongst other risk factors. Conventional management of thrombosis-based disorders includes the use of heparin, oral anticoagulants, and the preferred antiplatelet agent aspirin. Interestingly, aspirin was not intended to be used as an antiplatelet agent; rather, after being repurposed, it has become one of the most widely prescribed antithrombotic drugs. To this end, there have been several milestones in the development of antiplatelet agents in the last few decades, such as adenosine diphosphate receptor inhibitors, phosphodiesterase inhibitors, and GPIIb/IIIa inhibitors. However, given some of the limitations of these therapies, aspirin continues to play a major role in the management of thrombotic and cardiovascular disorders and is expected to do so for years to come.

## 1. Role of Platelets in Primary Hemostasis and Atherothrombosis

Since the mechanism of action of acetyl salicylic acid (aspirin) is based on platelets function, a complete knowledge of platelets physiology and pharmacology in hemostatic process is fundamental. Platelets were recognized as a distinct blood element in the late 19th century. Experiments performed *in vivo *by Bizzozero in 1882 demonstrated that platelets (and not white blood cells) were responsible for the formation of white clots at the sites of vascular injury in guinea pig micro vessels [1]. Mammalian platelets are enucleated cells arising from cytoplasmic fragmentation of megakaryocytes in the bone marrow and have a typical diameter of 2 to 3 *μ*m. Platelets circulate in a discoid form, and their average lifespan in humans is about 10 days [2]. Despite their lack of a nucleus, platelets contain a variety of mediators that regulate many processes from fighting microbial infection and triggering inflammation to promoting tumor angiogenesis and metastasis [3–6]. Nevertheless, the main function of platelets is to be essential mediators of the process of hemostasis and thrombosis. In fact, platelets do not interact with vascular walls under normal conditions, but when a blood vessel is damaged at its luminal side, platelets promptly adhere to the damaged subendothelial matrix, which contains several adhesive macromolecules like collagen, von Willebrand factor (vWF), laminin, fibronectin, and thrombospondin, to limit hemorrhage and promote tissue healing. 

In order to act as a hemostatic plug, relevant constituents for thrombosis are present both on the cell membrane and in the cytoplasm of platelets. The platelet membrane, which consists of a bilayer of phospholipids, contains membrane glycoproteins that interact with various ligands within the vessel wall or on other cells, through which the platelets adhere to the injured subendothelium and within each other. The plasma membrane of platelets is a highly specialized structure that contains a network of invaginations into the interior of the cell, connected to the exterior through small pores [7, 8], known as the open canalicular system, giving to the platelet a greater surface area than expected for such a small cell. Platelets contain a second channel system, derived from megakaryocyte smooth endoplasmic reticulum, known as the dense tubular system (DTS), which stores calcium and a variety of enzymes involved in platelet activation. Interestingly, the DTS system is not associated with the plasma membrane [9, 10]. Calcium is a critical factor for platelets function. It is the responsible of the dramatic changes in shape and ultrastructure that platelets undergo after the activation process, when platelets membranes become ruffled with cytoplasmic projections and the granules found inside the platelets are centralized and discharged [11, 12].

Accumulation of platelets at the site of vascular injury includes (1) an initiation phase involving platelet adhesion, (2) an extension phase that includes activation, additional recruitment, and aggregation, and (3) perpetuation phase characterized by platelet stimulation of the clot [13]. The initial phase of primary hemostasis is characterized by platelets adhesion and involves interactions between the glyproprotein (GP) Ib-IX-V receptor complex and the A1 domain of vWF in the exposed subendothelium [14, 15]. *Ex vivo *studies with human blood have demonstrate that GPIb-vWF is the primary adhesive interaction, initiating platelet adhesion at high shear rates (>1000 s^−1^) [16], as found in arterial microvessels or arterioles [17]. Analogous studies in mice (an increasingly studied experimental model of thrombosis) reveal comparable shear rate-dependence of GPIb-vWF-mediated adhesion occurring at higher shear rates [18]. This is because the initial adhesive interactions between platelets and the extracellular matrix depends on the local rheological conditions. Thus, at low shear rates (<1000^−1^ such in veins and larger arteries), platelets adhesion involves binding to collagen, fibronectin, and laminin. On the other hand, at higher shear rates (>1000^−1^, when a significant reduction in vascular cross-sectional area is present, such in small arteries and microvasculature, but also as may result from thrombus, atherosclerotic plaque, vasoconstriction, etc.), the interaction between the platelet surface receptor and GPI*α*-vWF interactions is critical to slow down the flow of platelets, allowing the establishment of additional bonds, leading to definitive arrest of platelets and subsequent thrombus formation [19, 20].

## 2. Platelet Adhesion Molecules

As previously mentioned, platelets contain a number of adhesion molecules, both on the plasma membrane and within their granules, which are relevant for hemostasis and thrombosis, as well as cell-cell and cell-subendothelial matrix interactions. The cellular localization and activation state of these molecules vary according to the state of platelet activation.

### 2.1. P-Selectin

(CD62P, *∼*140 kd) is the largest of the selectin family of adhesion molecules. It is contained primarily on platelet *α*-granules and in the Weibel-Palade bodies of endothelial cells. Under resting, unstimulated conditions, only little P-selectin is evident on the surface of platelets. However, following the activation of platelets (or endothelial cells), the membrane of the granule merges with the membrane of the cell, resulting in rapid expression of P-selectin on the cell surface [21, 22]. P-selectin surface expression is commonly used as a marker of platelet activation [23].

### 2.2. Glycoprotein Ib/IX/V (GPIb/IX/V)

This large glycoprotein receptor complex is the main platelet receptor for vWF and is composed of four distinct molecules [24]. Binding of vWF to GPIb initiates signal transduction events that lead to the activation of the platelet integrin GPIIb/IIIa, which becomes competent to bind vWF or fibrinogen to mediate platelet aggregation. Deficiency or dysfunction of the GPIb complex results in a bleeding disorder known as the Bernard-Soulier syndrome [25].

While this receptor is present constitutively on the platelet plasma membrane and vWF is normally present in plasma, binding of the receptor with its ligand induces alterations in blood flow and the resultant shear stress, with a subsequent conformational change in either or both components. This interaction is assessed *in vitro *using the antibiotic ristocetin instead of shear stress [26].

### 2.3. Glycoprotein IIb/IIIa (GP IIb/IIIa)

Platelet GP IIb/IIIa (also known as *α*IIb*β*3) is a heterodimeric transmembrane protein molecule composed of one *α* subunit (GP IIb) and a *β* subunit (GP IIIa), which belongs to the integrin family. This molecule is expressed constitutively on the plasma membrane as an inactive form in resting platelets. Upon platelet activation, the GP IIb/IIIa undergoes conformational changes, thereby gaining the ability to bind ligands [27]. Platelet GPIIb/IIIa can bind to fibrinogen as well as other ligands such as vWF, fibronectin, and vitronectin [28]. This molecule represents a major target for directed therapy in patients with thrombotic disorders [29].

### 2.4. Collagen Receptors

The **α**2*β*1 integrin and glycoprotein VI (GPVI, *∼*65 kd) are the primary collagen receptors, which play a prominent role in hemostasis. These receptors bind to specific sequences on collagen with different affinities. Platelet adhesion promoted by integrin *α*2*β*1 induces activation of platelet GPIIb/IIIa through the phospholipase C- (PLC-) dependent stimulation of the small GTPase Rap1b [30]. Platelet GPVI is expressed constitutively on the platelet plasma membrane and is also expressed on **α**-granules [31]. Following platelet activation, the surface expression of GPVI increases and intracellular expression decreases, consistent with their release from *α*-granules and incorporation into the plasma membrane. GPVI belongs to the immunoglobulin superfamily that contains two C2 immunoglobulin-like domains and an arginine residue in the transmembrane region that forms a salt bridge with the aspartic acid residue of the Fc receptor *γ*- (FcR*γ*-) chain [32]. Activation by collagen leads to phosphorylation of its immunoreceptor tyrosine-based activation motif (ITAM), leading to a sequence of events involving several adaptor proteins and resulting in phosphorylation and activation of PLC*γ*2 [33, 34]. GPVI mainly binds to collagen types that can form large collagen fibrils such as collagen type III. Absence of GPVI in humans is associated with a predisposition to bleeding [35].

## 3. Platelets Activation, Additional Recruitment, and Aggregation

Following the initial adhesion of platelets to the site of injury, platelets are activated by a process that occurs in 3 phases: (1) the interaction of agonists with their respective platelet receptors and receptor-mediated early platelet activation signaling, (2) the intermediate common signaling events, and (3) integrin activation. Platelet adhesion receptors are the key initiators of platelet activation at sites of vascular injury, where platelets become exposed to adhesive proteins in the matrix or on endothelial cells. Platelets are then activated by a number of agonists such as adenosine diphosphate (ADP) and collagen that are present at the sites of vascular injury. These agonists activate platelets by binding to the aforementioned receptors on the platelet surface described above. Occupancy of these receptors leads to a series of downstream events that ultimately increases the intracytoplasmic concentration of calcium ions, by release from intracellular stores, and by calcium influx through the plasma membrane [36]. Receptors coupled to G-proteins, such as those to ADP, thromboxane A_2_ (TXA_2_), and thrombin, activate phospholipase C*β*(PLC*β*), whereas receptors acting via the nonreceptor tyrosine kinase pathways such as collagen receptor GPVI preferentially activate phospholipase C**γ**(PLC**γ**) [36]. Activation of PLC*β* or PLC**γ** results in the production of two second messengers: diacylglycerol (DAG) and inositol trisphosphate (IP3). DAG mediates calcium influx, while IP3 liberates calcium from intracellular stores. Calcium influx may also be induced directly by certain agonists, such as ATP binding to the ligand-gated ion channel receptor, P2X1 [37].

As already pointed out, increased platelet-free calcium concentration results in a number of structural and functional changes of the platelet. Morphologically, the platelet changes dramatically from a disc to a spinysphere (a process called shape change). The granules in the platelet are centralized, and their contents are discharged into the lumen of the open canalicular system, from which they are then released to the exterior (i.e., the “release reaction”). The increase in platelet calcium stimulates membrane phospholipase A_2_ activity, which liberates arachidonic acid (AA) from membrane phospholipids. This AA is converted to an intermediate product prostaglandin H_2_ (PGH_2_) by the enzyme cyclooxygenase 1 (COX-1). PGH_2_ is further metabolized to TXA_2_ by thromboxane synthase [38]. TXA_2_ is a potent activator of platelets. The long membrane projections brought about by shape-change reaction allow the platelets to interact with one another to form aggregates. Shape change is mediated by the platelet cytoskeleton, composed by an organized network of microtubules and actin filaments and a number of associated proteins, linked to a variety of platelet signaling molecules [39]. Platelet shape change results in extensive reorganization of the cytoskeleton network, polymerization of actin, and myosin light chain phosphorylation [39–42]; these responses vary in a time- and stimulus-dependent manner.

## 4. Amplification of Activated PlateletsSignal Transduction: Receptor-MediatedEarly Platelet Activation Signaling andIntermediate Common Signaling Events

Following platelet binding to the injured vessel wall and subsequent signaling to the platelet cytoplasm, a controlled release reaction takes place. Platelet granules fuse with the outer membrane and empty their content into the local environment, filling it with a multitude of bioactive molecules. Their para- and autocrine nature causes preliminary signals to quickly feedback into the process by increasing activation of nearby platelets in both number and magnitude, thereby evoking secondary secretion, resulting in a drastic amplification of the platelet activation process [43].

In platelets, three types of granules are distinguished: **α** granules, dense granules (**δ**), and lysosomes (**γ** granules). Alpha (**α**) granules are the largest (*∼*200–400 nm) and most prevalent and heterogeneous platelet granules [44, 45]. These granules are responsible for a variety of effects like primary hemostasis, coagulation, inflammation, angiogenesis, wound healing, and others. The definitive proof of the importance of *α* granules in hemostasis came from the evidence of the consequences of its deficiency. Platelet *α*-granule deficiency is a rare inherited disease, known as the gray platelet syndrome (GPS). This illness is associated with quantitative and qualitative platelet dysfunction and a bleeding predisposition [46]. In GPS, proteins endogenously synthesized by megakaryocytes or endocytosed by platelets fail to enter *α*-granules of platelets due to abnormal formation of *α*-granules during megakaryocytic differentiation. This results in continued release of *α*-granule contents such as growth factors and cytokines into the bone marrow resulting in fibrosis (myelofibrosis) [46].

The **α** granules contain the majority of platelet factors involved in hemostasis and thrombosis. These include large polypeptides such as thrombospondin, P-selectin, platelet factor 4, and *β*-thromboglobulins. *α*-granules also contain a variety of adhesion molecules involved in platelet-vessel wall interaction such as fibronectin and vitronectin. The membrane of *α*-granules contains several proteins that are also expressed on the platelet cell membrane such as GPIb complex, GPVI and GP IIb/IIIa [47]. Besides the release of proteins important in primary hemostasis, **α** granules contribute to secondary hemostasis. In fact, coagulation factors also reside in the **α** granules (Factors V, XI, and XIII, fibrinogen, vWF, and high molecular weight kininogens). Controlled fibrin formation stabilizes platelet aggregates in environments of high shear stress, and stabilization of fibrin threads is assured through the simultaneous release of fibrinolysis inhibitors like plasminogen activator inhibitor-1 [48].

### 4.1. Dense Granules Release and Amplification of Platelet Signaling

Platelet dense granules are the smallest granules (*∼*150 nm) and appear as dense bodies on electron microscopy due to their high calcium and phosphate content [9]. In addition, they contain high concentrations of adenine nucleotides and serotonin. The nucleotide ADP is more abundant and more potent than serotonin. It interacts with two biochemically related purinergic G-coupled receptors that evoke distinct reactions through separate signaling pathways. Binding of ADP to P2Y1 causes platelet shape change and aggregation through G_q_-mediated phospholipase C-*β*2 [49, 50]. The shape change reaction precedes aggregation and increases the external platelet surface area, helping contacts with nearby cells and matrix molecules. The binding of ADP to its second receptor (i.e., P2Y12) induces the coupling to G*α*_1_ and decreases cAMP [51]. The P2Y12 receptor also stimulates surface expression of P-selectin and secretion of TXA_2_ [52]. This latter, short-lived prostanoid is of major importance for signal amplification through binding with the thromboxane/endoperoxide receptor (abbreviated as TPR), which, in turn, activates G_q_ and G_13_, thereby closing the loop [53]. TXA_2_ is produced de novo and binds receptors TPR_*α*_ and TPR_*β*_; however, its effects in platelets are mediated primarily/solely through TPR_*α*_ [54]. Both ADP and TXA_2_ are secreted from adherent platelets and contribute to the recruitment of circulating platelets and promote alterations in platelet shape and granule secretion, thus platelet activation is amplified and sustained during the extension phase. 

The importance of dense granules to normal hemostasis is highlighted by the bleeding disorder produced in patients with deficiency of these granules. Platelet dense granule deficiency has been identified in two rare human conditions associated with predisposition to bleeding: Hermansky-Pudlak syndrome (HPS) and Chediak-Higashi syndrome [55]. HPS is defined by pigment dilution (affecting skin, hair, and eyes), resulting in oculocutaneous albinism and platelet storage pool deficiency due to deficiency of dense granules. HPS is due to mutations in genes that mostly function in membrane and protein trafficking. There are eight known human HPS genes, each resulting in specific clinical variants of HPS [56]. Mouse strains that are deficient in orthologous genes also have been characterized and have a bleeding diathesis [57]. Chediak-Higashi syndrome is a rare autosomal recessive disorder characterized by oculocutaneousalbinism, lymph node enlargement, hepatosplenomegaly (liver and spleen enlargement), and recurrent infections [55, 58]. Platelet aggregation studies are consistent with deficiency in the storage pool of dense granule substances and suggest that this granule defect has an influence on the release mechanism of other granule constituents[59].

Lysosomes represent the third category of platelet granules, with a size intermediate between **α**- and dense granules (*∼*200–250 nm); they contain an intraluminal acidic pH with hydrolytic enzymes active towards a number of substrates including constituents of the extracellular matrix [9, 60]. While the functional role of platelet lysosomes is less well understood than that of **α**- and dense granules, lysosome release has been postulated to contribute to regulation of thrombus formation and remodeling of the extracellular matrix [9]. Whereas dense body contents are easily secreted, a granule secretion only occurs with powerful activating agents such as thrombin or high doses of collagen. In addition to the contents of the three types of granules, and as stated previously, platelets also produce and secrete pharmacologically active substances such as TXA_2_ and the platelet-activating factor (PAF) during their activation and aggregation, which establish a positive feedback system.

## 5. Mechanisms of Platelet Activation: Platelets Receptors

Platelets can be activated by a variety of physiological and pharmacological agents. All these agonists are believed to exert their effect through the interaction with specific receptors on the platelet plasma membrane. All of the agonist receptors, which interact/couple with guanine nucleotide-binding regulatory proteins (G-proteins) or G-proteins that have been identified to date, consist of a single polypeptide with an extracellular N-terminal domain which serves as the activator-binding domain, a seven hydrophobic transmembrane domains, and an intracellular C-terminal domain which is in connection with cytoplasmic second messenger generating enzymes. The primary effects of these agonists are often enhanced by secondary effects attributable to the synthesis of TxA_2_ from released AA and to the secretion of ADP [61]. 

The best known platelet receptors are as follows.

1. *vWF* is present in the subendothelium and in platelets. Upon binding to subendothelial components, especially type VI collagen, vWF undergoes a conformation change which enables it to bind to platelet GP Ib. This is one of the major sialogly coproteins of the platelet membrane consisting of two disulfide-linked subunits. The interaction between vWF and GP Ib results in platelet activation and in the generation of an intraplatelet signal necessary to activate GP IIb/IIIa [62], thereby leading to platelets spreading and irreversible platelet adhesion, which can resist the high shear forces in blood circulation.
2. *Thrombin Receptor: *thrombin is a very powerful platelet stimulus, causing shape change, aggregation, and secretion from dense granules, *α* granules, and lysosomes. Its receptor is a member of the G-protein coupled seven transmembrane family. It is cleaved by thrombin between arginine 41 and serine 42, which results in its activation. After thrombin activation, desensitization to further thrombin activation occurs with internalization of the thrombin receptors in endosomes, where three quarter of the receptors are transferred to lysosomes and degraded [63].
3. *Collagen Receptor: *GPVI is the major platelet collagen receptor to mediate cellular activation, which is a prerequisite for efficient adhesion, aggregation, degranulation, and coagulant activity on the matrix protein [64–66]. GPVI (62 kDa) is a type I transmembrane receptor expressed exclusively in platelets and megakaryocytes [67]. GPVI has only a low affinity for collagen, which makes it similar to GPIb*α*, unable to mediate adhesion by itself. It is now widely accepted that although GPVI serves as a receptor, that is, essential for platelet activation and aggregation by collagen, it also contributes to firm adhesion. Thus, both collagen receptors act synergistically, reinforcing each other's activity ensuring optimal platelet adhesion and activation by collagen [68].
4. *ADP Receptors: *there are two known subtypes of ADP receptors on human platelets P2Y1 which couples with G*α*_q_ and contributes to initial aggregation, and P2Y12 receptors which are coupled to G*α*_1_ and decreased cAMP. P2Y12 also induces surface expression of P-Selectin and TxA_2_ [51]. A new receptor has been characterized in mice and called P(2T). In those animals, sustained ADP-induced aggregation requires coactivation of P2Y1 and P(2T) receptors. Studies using AR-C69931MX, a selective P(2T) receptor antagonist and novel antithrombotic agent, were performed lately to further characterize this receptor. These studies have confirmed that the P(2T) receptor plays a central role in amplifying platelet responses [69].

### 5.1. Platelet Role in Clot Formation

Activated platelets also provide an efficient catalytic surface for the assembly of the enzyme complexes of the blood coagulation system [70–72], known as secondary hemostasis. The classic description of coagulation involves a cascade model consisting of two distinct pathways: the extrinsic, or tissue factor pathway and the intrinsic pathway. These are now viewed in terms of overlapping phases of initiation, amplification, and propagation. The coagulation system consists of a number of serine proteases, cofactors, calcium, and cell membrane components, and their reactions represent highly complex interactions, subject to regulation at a number of levels that eventually will lead to the formation of cross-linked and insoluble interconnecting networks of strands. This particular characteristic of platelets makes them an important target for drug discovery and applications in the field of thrombosis pathologies.

The characteristics of the procoagulant activity on the platelet surface resemble those of synthetic phospholipid surfaces. To be active in coagulation, the phospholipid surface requires a net negative charge (provided by phosphatidylserine, phosphatidylinositol, or phosphatidic acid), an optimal degree of unsaturation of the acyl chains, and an appropriate size [73]. In resting platelets, phosphatidylserine and other anionic phospholipids are located on the inner aspect of the membrane bilayer [74]. Following platelet activation with thrombin, collagen, or shear stress, phosphatidylserine moves from the inner to the outer leaflet of platelet plasma membrane. This movement is associated with an increase in the activation of prothrombinfactor II and factor X [75] and with the appearance of high-affinity binding sites for factors Va and VIIIa.

The importance of the exposure of anionic phospholipid for hemostasis *in vivo *is exemplified by Scott syndrome, a rare bleeding disorder first described in 1979 by Weiss et al. as an isolated deficiency of platelet procoagulant activity [76]. Platelets of patients' carriers of this syndrome have a reduced number of binding sites for factor Va and VIIIa and did not promote prothrombin or factor X activation. Furthermore, following platelet activation with thrombin and collagen, they present a marked decrease in the exposure of anionic phospholipid at the platelet surface as compared with normal platelets [77]. The clinical features of these patients illustrate the importance of platelet procoagulant activity in secondary hemostasis. Externalization of anionic phospholipid in platelets is accompanied by the generation of phosphatidylserine-rich microvesicles [78], suggesting that microvesicles and anionic phospholipid exposure are linked events.

Several findings suggest that in addition to its role in normal hemostasis, platelet microvesiculation may contribute to the prothrombotic tendencies observed in several diseases. Platelet-derived microvesicles have been detected in the circulation in patients with disseminated intravascular coagulation [79], heparin-induced thrombocytopenia [80], the antiphospholipid antibody syndrome [81], transient ischemic attacks [82], and thrombotic thrombocytopenic purpura [83], conditions associated with either arterial, venous, and/or microvascular thrombosis. These associations suggest that while they may be necessary for normal hemostasis, elevated microvesicle concentrations could predispose to thrombosis. Thus, conditions that increase the production or decrease the clearance of microvesicles are expected to increase the incidence of thrombosis. 

The microvesicles bind activated platelets in thrombi through a molecular bridge between P-selectin glycoprotein ligand-1 (PSGL-1) on microvesicles and P-selectin on platelets. These microvesicles selectively enriched in both tissue factor and PSGL-1 fuse with activated platelets, transferring tissue factor to the platelet membrane [84]. Failure of this hemostatic mechanism may explain the ability of agents that block the PSGL-1-P-selectin interaction to significantly inhibit experimental thrombosis [85]. The role of tissue factor-bearing microvesicles in normal hemostasis is an active area of investigation.

## 6. Clinical Aspects of Thrombosis and Aspirin Use in Cardiovascular Disease

Clearly, platelets play a key role in normal hemostasis and in the pathogenesis of atherothrombotic disease. Because both pathological and physiological functions of platelets are due to the same mechanism, it is difficult to separate the therapeutic benefits from harmful effects. The ultimate goal of any antithrombotic treatment is to increment the efficacy and reduce the risks to the patient. For those reasons, antiplatelet therapy has become a useful means of preventing acute thromboembolic artery occlusions in cardiovascular disease. The rationale for this is an enhanced activity of circulating platelets and an inhibition of the release of platelet-derived vasoactive mediators, probably due to endothelial dysfunction. In spite of the progress in the field of platelets function, aspirin is still considered the leading drug in the field. Below, we discuss the basis for the use of aspirin as the antiplatelet drug of choice for long-term oral treatment, specifically for secondary prevention of myocardial infarction, and also as a suitable basic but not maximally efficient drug in percutaneous transluminal coronary angioplasty (PTCA), and platelet activation during clot lysis.

### 6.1. Aspirin: Pharmacology of Antiplatelet Activity

The benefits of antiplatelet therapy for the prevention of thrombotic events in cardiovascular diseases are evident. Statistical studies have shown that secondary prevention by antiplatelet agents reduces the risk of nonfatal myocardial infarction (MI) and stroke by 25% to 30%, and the rate of vascular death by about 15%, resulting in a significant reduction in overall mortality [86]. These data demonstrate that (1) blood platelets, circulating in an activated state, are important determinants of arterial thrombus formation and vessel occlusion and (2) these processes can be antagonized by appropriate antiplatelet therapy. However, it is also clear that one of the major problems in clinical use will be the separation of the antithrombotic efficacy of antiplatelet agent from interference with the physiological platelet function in hemostasis. 

Antiplatelet drugs are classified on the basis of their site of action, that is, drugs that inhibit (i) platelet adhesion, (ii) platelet activation, (iii) platelet aggregation, and (iv) platelet-mediated links with inflammation [87]. Aspirin belongs to the group of drugs that inhibit platelet activation. As seen before, platelet activation can be blocked by inhibited the TXA_2_ pathway, ADP pathway, thrombin pathway, and phosphodiesterase (PDE). Aspirin meets its effects by inhibiting the TXA_2_ pathway in a dose-dependent manner. 

Low-dose (75–81 mg) aspirin inhibits cycloxygenase-1 (COX-1) in such a way that only TXA_2_ production is inhibited and not of PGI_2_. Gastrointestinal tract (GIT) bleeding, drug interactions, and resistance are major drawbacks of aspirin. To avoid these drug reactions, work is ongoing for new strategies such as inhibition of thromboxane synthetase enzyme and blockade of TPRs receptors. TXA_2_ synthetase is not much efficacious clinically, because blockade of this enzyme results in accumulation of endoperoxide precursors which are themselves platelet TPR agonists [88].

It was Vane, a researcher, that in 1971 discovered the mechanism of the analgesic, antipyretic, and anti-inflammatory actions of aspirin [89]. For his experiments, the supernatant of a broken cell homogenate from guinea pig lung was incubated with AA and with different concentrations of aspirin, indomethacin, or sodium salicylate. After 30-minute incubation at 37°C, prostaglandin (PG)F_2*α*_  generation was estimated by bioassay on rat colon. Vane found a dose-dependent inhibition of PGF_2*α*_ formation by all three drugs, indomethacin being the most potent and sodium salicylate the least. Three control drugs, morphine, hydrocortisone, and mepyramine, had no effect on prostaglandin synthesis. Two other reports in the same issue of Nature lent support to this finding and extended it considerably. Firstly, Smith and Willis [90] were investigating the effects of aspirin on platelet behavior. They found that thrombin added to a suspension of platelets *in vitro *to which various concentrations of aspirin had been added also released lower levels of prostaglandins and caused less aggregation. Secondly,Ferreiraand his colleagues [91] demonstrated that aspirin and indomethacin blocked the release of prostaglandins from a perfused, isolated dog spleen subjected to sympathetic nerve stimulation. These experiments explained why all the aspirin-like, or nonsteroid anti-inflammatory drugs (NSAIDs) as they became known, shared the same pharmacological actions: anti-inflammatory, analgesic, and antipyretic and the same side effects of damage to the stomach mucosa, toxicity to the kidney, and inhibition of platelet aggregation. There was already evidence suggesting that PGE_1_ was a pyretic agent in several species [92] and that PGE_2_ mimicked the inflammatory response when injected intradermally [93], leading to speculations that prostaglandins might be responsible, at least in part, for the genesis of fever and inflammation and that aspirin-like drugs might owe their therapeutic activity to their ability to prevent prostaglandin biosynthesis.

In 1976, an enzymatically active COX-1 or prostaglandin endoperoxide synthase (PGES) was isolated [94]. The enzyme was cloned and its structure elucidated in 1988 [95]. This membrane-bound hemo- and glycoprotein with a molecular weight of 71 kd is a constitutive enzyme found in greatest amounts in the endoplasmic reticulum of most mammalian cells [96].

COX converts AA, a *ω*-6polyunsaturated fatty acid PUFA, to prostaglandin H_2_ (PGH_2_), the precursor of the series-2 prostanoids. The enzyme contains two active sites: a heme with peroxidase activity, responsible for the reduction of PGG_2_ to PGH_2_, and a cyclooxygenase site, where AA is converted into the hydroperoxyendoperoxide PGG_2_. The reaction proceeds through H atom abstraction from AA by a tyrosine radical generated by the peroxidase active site. Two O_2_ molecules then react with the arachidonicacid radical, yielding PGG_2_ [97].

The COX active site is a long hydrophobic channel, and Picot et al. [98] presented evidence that most of the aspirin-like drugs such as flurbiprofen inhibit COX by excluding arachidonate from the upper portion of the channel. Tyrosine (Tyr) 385 and serine (Ser) 530 are situated at the apex of the long active site. Aspirin inhibits COX by acetylation of the hydroxyl group of Ser 530, thereby excluding access for AA to Tyr 385 by steric hindrance [99]. This covalent bond results in an irreversible inhibition of COX unlike the reversible inhibitory action of other NSAIDs. Aspirin acetylates COX in platelets in the presystemic circulation, where there is a high concentration of aspirin in the portal vein before its metabolism by the liver. This inhibition of platelet function occurs with very low doses of aspirin which have no systemic effects and provides the basis for the use of daily 75 mg doses of aspirin in the prevention of heart attacks and strokes. It is irreversible and platelets lose their ability to aggregate until new platelets are formed, which is within 8 to 11 days in humans [100].

In 1991, a second COX encoded by a different gene from the first COX was discovered [101]. COX-2 is an inducible enzyme that is expressed in response to inflammatory stimuli released from bacteria such as lipopolysaccharides, cytokines released from macrophages like interleukin-1, mitogens, and growth factors. In terms of their molecular biology, COX-1 and COX-2 are of similar molecular weight, approximately 70 and 72 kd, respectively, and having 65% amino acid sequence homology and near-identical catalytic sites. The most significant difference between the isoenzymes, which allows for selective inhibition, is the substitution of isoleucine at position 523 in COX-1 with valine in COX-2. It makes the active site of COX-2 slightly larger than that of COX-1. The smaller Val523 residue in COX-2 allows access to a hydrophobic side pocket in the enzyme (which Ile523sterically hinders). Drug molecules, such as DuP-697 and the “coxibs”, which are derived from it, bind to this alternative site and are considered to be selective inhibitors of COX-2 [102, 103]. Aspirin still acetylates serine 516 in COX-2, but due to the larger size of the catalytic channel, AA is able to “squeeze past” the acetylated structure and form 15- R-hydroxyeicosatetraenoic acid (15-R-HETE) [104]. The discovery and characterization of COX-2 explained the variation in the relative potency of the therapeutic actions of the NSAIDs, compared to their activity in producing side effects.

A new COX-1 variant named COX-3 present in high concentrations in dog brain was identified in 2002 [105]. COX-3 is a splice-variant of COX-1 that retains the intron-1 gene sequence at the mRNA level which encodes a 30 amino acid sequence inserted into the N-terminal hydrophobic signal peptide of the enzyme protein. The variant enzyme was more sensitive to inhibition with paracetamol than either COX-1 or COX-2, and it was also more sensitive to the inhibitory action of nonselective NSAIDs including aspirin. COX-3 enzyme protein has been identified by Western blotting in human tissues, and it is thought to be involved in the hypothermic and analgesic action of paracetamol in mice, since paracetamol penetrates easily into the central nervous system [106].

In brief, aspirin irreversibly inhibits COX-1 by acetylating serine 529, thereby inhibiting the production of TXA_2_, a promoter of platelet aggregation, and prostaglandin I_2_, a potent inhibitor of platelet aggregation and powerful vasodilator, in platelets and vascular endothelial cells, respectively. In the absence of protein synthesis in platelets, TXA_2_ inhibition persists for the lifetime of the platelet compared with vascular endothelial cells, which recover COX-1 activity shortly after exposure to aspirin. For over 50 years, aspirin has been the basis of antiplatelet therapy, and it remains so today [107]. Aspirin may also be of benefit in the primary prevention of cardiovascular events, but the effect is more modest, and its recommendation is highly debated due to the fact that ischemic benefit may be offset by bleeding complications. Although aspirin is a cost-effective therapy, it is a weak antiplatelet agent [108], and a considerable number of patients continue to experience atherothrombotic complications, especially high-risk patients, such as those presented with acute coronary syndrome (ACS) or undergoing percutaneous coronary intervention (PCI).

Despite its universal use, the optimal dose of aspirin for efficacy and safety remains unclear. Previous studies agree that aspirin at ≥300 mg is similar to aspirin 75–100 mg/day for the prevention of major vascular events and that higher doses increase the risk of bleeding [108].

Recently, the CURRENT-OASIS 7 trial, a randomized head-to-head comparison of higher-dose (≥300 mg/day) versus low-dose (≤75–100 mg/day) aspirin in patients with ACS demonstrate similar outcomes from efficacy, without a difference in the risk of major bleeding complications [109]. 

On the other hand, one of the limitations for the use of aspirin is the emergence of resistance. Some of the theories which surround the reasons for aspirin resistance include potential isoprostane interaction with aspirin treatment and emerging pharmacogenomic data in which variants and polymorphisms of essential role players in aspirin pharmacology change its efficacy. It is important to note that isoprostanes can be produced at much higher levels *in vivo* than the classical prostaglandins/thromboxanes [110]. Furthermore, it is known that the *in vivo *levels of isoprostanes can be enhanced by the presence of vascular disease compounded with traditional aspirin therapy, which has also been implicated with increasing isoprostane levels [110, 111]. Hence, the theory on resistance involves the theory that increased isoprostane levels, among other factors, may contribute to aspirin resistance [112], particularly because isoprostanes have the capacity to activate platelet TPRs [110]. Thus, by inhibiting COX-1, and blocking TXA_2_ synthesis, aspirin appears to facilitate increased isoprostane production, by making more AA/substrate available for their production, which, in turn, may alter the antithrombotic effects of aspirin itself, rendering it less effective.

Genetic polymorphisms of platelet receptors also show impact on drug resistance. Some of the common receptors that have polymorphisms which can affect platelet reactivity include a PLA_1_/A_2_ single-nucleotide polymorphisms (SNPs) of GPIIb/IIIa (fibrinogen integrin), C807T SNP of GPIV (collagen integrin), and variants A, B, C, and D of GPIb (von Willebrand factor integrin) which comprise different repeating portions in the amino acid sequence which makes up the receptor [113]. These variants and different polymorphisms can contribute to resistance or reduced efficacy of certain drugs such as aspirin. Specifically, it has been shown that the PLA_1_/A_2_ polymorphism was significantly associated with aspirin resistance and have diminishing effect in the presence of cardiovascular disease [114]. In addition, other polymorphisms and enzymes are implicated in potential aspirin resistance including, UDP-glucuronosyltransferase UGT1A6 enzyme, cytochrome P450 CYP2C9 metabolic enzyme, xenobiotic ACSM2, and PTGS1/2 which are responsible for COX1/COX2 coding [115]. It is important to mention, however, that extrinsic factors are also important in aspirin resistance. Recent studies have shown that specific COX1 A-842G, C50T, and GPIIIa PLA_1_/A_2_ genetic polymorphisms are frequently observed in Caucasians and were not observed in patients from mainland China. Data such as this would imply that many people in the general population may have genetic character which makes them poor aspirin candidates. Importantly, the presence GPIIb/IIIa PLA_1_/A_2_ polymorphism seems to be related to an attenuated antiplatelet effect during aspirin treatment, if compared to individuals that do not present such a genetic polymorphism [116].

## 7. Aspirin in Primary Prevention of Cardiovascular Complications

Although the use of aspirin in secondary prevention against cardiovascular complications is widely accepted, there is controversy over the daily use of aspirin for primary prevention of cardiovascular disease (CVD) in persons who have yet to demonstrate clinical evidence of CVD. Many studies have been performed in order to overcome this issue, with different conclusions that have not resulted in a common accepted protocol. 

The American Heart Association recommends the daily use of aspirin (71–326 mg) indefinitely in all patients with known CVD for secondary prevention, unless contraindicated [117]. In 2009, the U.S. Preventive Services Task Force (USPSTF), based on the results presented in different trials, concluded that aspirin is in fact effective for primary CVD prevention [118], indicating that aspirin for primary prevention should be recommended when benefits outweigh risks. The USPSTF found good evidence that aspirin use decreases myocardial infarctions in men from 45 to 79 years of age with a 10 year CVD risk but without previous cardiovascular events and in women from 55 to 79 years of age with a 10-year stroke risk that have not yet experienced cardiovascular events. Several online calculators are available to help physicians determine these risks [119]

Recent studies, however, have questioned whether the benefits of daily aspirin for primary CVD prevention outweigh the risks of gastrointestinal (GI) and intracerebral (IC) hemorrhage [108, 120, 121]. Currently, two large, international randomized controlled trials (*n* > 10,000) are investigating the benefit of daily aspirin therapy for primary CVD prevention in select populations: the ASCEND trial (a study of cardiovascular events in diabetes) and the ASPREE trial (aspirin in reducing events in the elderly), but conclusive results from these trials should be available within the next five years. 

In addition, a recent meta-analysis of six primary prevention trials in women found that the cardiovascular benefit of aspirin is frequently outweighed by the risks of bleeding. In this study, there was a 12% proportional reduction in serious vascular events (0.51% aspirin versus 0.57% control per year; *P* = 0.0001), primarily owing to a reduction in nonfatal myocardial infarction (MI), but there was not significant reduction in events such stroke or vascular mortality [108]. Those results correlate to those presented in a previous trial involving 39,876 relatively healthy women of 45 years and older. This study suggested that daily aspirin therapy may not decrease in a highly significant level the risk of acute myocardial infarction in women although there was observed a 17% decreased risk of stroke [122], showing that the use of aspirin as primary CVD prevention is dependent not only on the age but also on the gender of the patient. 

Aspirin for primary CVD prevention should be avoided in persons who have had a GI or cerebral bleeding episode and in those who are at risk of bleeding problems (e.g., bleeding disorder, severe liver disease, thrombocytopenia, and concomitant anticoagulant therapy). For persons with an aspirin-induced bleeding ulcer, aspirin use in combination with a proton pump inhibitor may be safely restarted after the ulcer has healed [123]. A randomized controlled trial found that in those taking low-dose aspirin who had GI bleeding, continuous aspirin therapy may increase the risk of recurrent GI bleeding but potentially reduces cardiovascular and cerebrovascular mortality rates [124]. Eradication of Helicobacter pylori decreases the risk of recurrent GI bleeding in those taking low-dose aspirin [125], while others studies found that enteric-coated or buffered aspirin did not decrease the risk of GI toxicity [126].

Since the absolute risk of CVD is much lower among patients being treated for primary prevention, some clinicians prefer to be more cautious and reserve aspirin therapy only for those who will clearly benefit, such as those with established disease. To help determine the efficacy of aspirin in primary prevention among patients with risk of CVD, the results of ASPREE and aspirin to reduce risk of initial vascular events (ARRIVE) trials are awaited. 

At present, the factor most strongly associated with appropriate aspirin use is a conversation between the patient and the physician [127]. The National Committee for Quality Assurance has proposed that health plans measure their members' use of aspirin, as well as the extent to which physicians discuss aspirin use with their patients. Determining patients' CVD risk and discussing appropriate aspirin use with them should be a priority for all physicians in order to implement an adequate prevention of cardiovascular diseases for each specific patient [119].

## 8. The Future of Antiplatelet Therapy in Cardiovascular Diseases

Since TXA_2_ is the primary product of COX-1-dependent metabolism of AA, whose biological actions are mediated through the TXA_2_ receptor (TPR), and because of the limitation associated with aspirin use, including severe gastrointestinal toxicity, bleeding complications, potential individual response, variability, and poor efficacy in some cardiovascular diseases and procedures, new interest has been focused towards additional TXA_2_-associated drug targets, in particular TXA_2_ synthase (TS) and the TPR. Previous attempts to develop TPS inhibitors and TPR antagonists have failed mainly due to poor pharmacodynamic properties. Persistent and complete platelet inhibition, as attained with aspirin, has not been achieved by these compounds. Despite the lack of clinical success, TS/TPR remains an attractive target, because other antiplatelet agents and anticoagulants are associated with comparable or greater risks. In fact, the American Heart Association (AHA) has recently published a scientific update for clinicians, based on new data from randomized controlled trials of COX-2 selective inhibitors [128], reinforcing previous warnings regarding drugs like rofecoxib, which was withdrawn in 2004 due to a high risk of heart attack and stroke, or celecoxib which in the Adenoma Prevention with Celecoxib clinical trial was suspended by the National Institutes of Health, because of increased cardiovascular events within the participants. At the end of 2004, the FDA issued a public health advisory summarizing the agency's recent recommendations for the use of the NSAID products Vioxx, Bextra, Celebrex, and Naproxen [129, 130]. The confirmation of the risk of those drugs together with the complexity to follow up all the recommendations for the safe use of such medications supports the superiority of TXA_2_ or TPR inhibitors in terms of clinical safety and efficacy, which remain as open questions that are the focus of current research [131].

In summary, low-dose aspirin is associated with a 25% reduction of secondary MI and stroke and 30% reduction on primary MI [86]. Aspirin inhibits COX-1 and at higher concentrations COX-2. Secondary effects of platelets inhibition, including reduced reactive oxygen species ROS, inflammatory cytokine, growth factor generation, and improved endothelial function, likely contribute to the overall beneficial effect. However, the reduced risk of arterial thrombosis cannot be dissociated from an increased risk of bleeding complications. This is particularly problematic in the upper gastrointestinal (GI) tract and in the brain. For secondary prevention, the benefit from aspirin outweighs the risk, but in low-risk populations, the risk of intracranial bleeds and serious GI adverse reactions is numerically balanced with the benefit [132]. However, aspirin is the primary antiplatelet aggregation drug used at present in combined therapies aimed to prevent further thrombotic events associated with a primary disease that presents high risk of embolic processes that could result in fatal outcomes.

## 9. Antiplatelet Therapy in Neurological Diseases

Anticoagulant treatment in neurology aims to reduce the risk of stroke as consequence of acute or chronic cardiac diseases. Transient ischemic attacks are a good indicator of the need for prophylactic antiplatelet/anticoagulant therapy. About 30% of patients with stroke have a history of transient ischemic attacks and proper treatment of the attacks is an important means of prevention. The incidence of stroke does not relate to either the number or the duration of individual attacks, but is increased in patients with hypertension or diabetes. The risk of stroke is highest in the month after a transient ischemic attack, particularly in the first 48 hours, and progressively decreases thereafter [133]. 

An important cause of transient cerebral ischemia that can be prevented by using the appropriate antiplatelet therapy is embolization. In many patients with these attacks, a source is readily apparent in the heart or a major extracranial artery to the head. Cardiac causes of embolic ischemic attacks include atrial fibrillation, rheumatic heart disease, mitral valve disease, infective endocarditis, atrial myxoma, and mural thrombi complicating myocardial infarction. Patients with high cholesterol levels may present atherosclerotic changes in the region of the carotid bifurcation extracranially. Also, patients with acquired immunodeficiency virus (AIDs) have an increased risk of developing transient ischemic deficits or strokes. 

Less common abnormalities of blood vessels may cause transient ischemic attacks. Furthermore, fibromuscular dysplasia, atherosclerosis of the aortic arch, and inflammatory arterial disorders such as giant cell arteritis, systemic lupus erythematosus, polyarthritis, and granulomatous angiitis are also risk factors for transient ischemic deficit or stroke as well as hematologic hyperviscosity syndromes [133]. 

Because of the elevated number of factors that can induce stroke, to find a balance in long-term antiplatelet therapy is important. Guidelines recommend an early initiation of antiplatelet/anticoagulant drugs when there is a cardiac source of embolization. Since comparative studies on the best starting dose for initiating aspirin therapy to achieve a rapid antiplatelet effect do not exist [134], approved protocol indicates initial treatment with warfarin. The target is an INR of 2.0 to 3.0 (laboratory measurement of prothrombin time). If the INR is within the range expected, the CT scan shows no evidence of hemorrhage, and the cerebrospinal fluid is clear, antithrombotic treatment may be started without delay, including aspirin treatment [133].

The anticoagulant approach after an ischemic attack or a stroke cannot be transferred directly from the antiplatelet treatment of coronary or peripheral disease to the treatment of patients with acute stroke. This is because of the heterogenic stroke cases and the bleeding vulnerability of the brain after acute cerebrovascular ischemia [135]. When treating acute stroke patients, neurologists try to reduce the risk of recurrent thromboembolic events but at the same time fear an excessive risk of cerebral bleeding. Data of the IST and CAST study groups showed an insignificant increase of hemorrhagic stroke with aspirin doses of either 160 mg or 300 mg and no elevated net hazard in patients who were inadvertently randomized after a hemorrhagic stroke [125]. The current guidelines recommendations on aspirin dosages for acute stroke patients are based on these two studies. At present, bedside testing of the antiplatelet effect of aspirin has become available using impedance aggregometry. In fact, studies evaluating dose-time response relationship of different aspirin dosages in healthy volunteers have been recently performed [136, 137]. Those studies have showed that the antiplatelet effect differs significantly depending on aspirin starting dosages (500 mg iv, 500 mg po, 200 mg/day/2 days, and 100 mg/day/5 days). Also, a high inter- and intraindividual variability of antiplatelet therapy, with a loading dose after the stroke of 500 mg aspirin IV, used to ensure an early platelet inhibitory effect in acute stroke patients. However, there are not enough conclusive clinical studies to define the best way and time to initiate antiplatelet therapy with aspirin. 

Lowering the risk levels of clot formation with antiplatelet/anticoagulant therapy is especially important when atrial fibrillation is the main factor that can induce embolism and stroke. Using pooling data from 5 prevention trials in atrial fibrillation, the overall annual rate of stroke was 4.5% in the control group compared with 1.4% in warfarin group [138], equal to a total reduction of 68% for ischemic stroke attributable to warfarin [139]. Warfarin efficacy was consistent across all the studies and subgroups of patients, and balanced against minimal changes in the annual rate of major hemorrhage, which was defined as intracranial bleeding or bleeding requiring hospitalization or 2 units of blood. This pooled analysis reported annual rates of major bleeding of 1% in controls and 1.3% in warfarin-treated patients. 

In contrast, the efficacy of aspirin for stroke prevention in AF was less consistent. The Second Copenhagen Atrial Fibrillation, Aspirin, and Anticoagulant Therapy (AFASAK 2) study showed a nonsignificant decrease in risk of stroke with 75 mg aspirin, while the Stroke Prevention in Atrial Fibrillation (SPAF) study found a significant decrease of 44% associated with 325 mg aspirin [136, 137]. The investigators found identical rates of major bleeding in the control (1.0%) and the aspirin group (1.0%). A later meta-analysis of aspirin therapy versus placebo found a 19% reduction in the overall incidence of stroke, with no evidence favoring one dose of aspirin over another [140]. Since then, many trials have been performed including patients with persistent or permanent AF. Anticoagulation was found to reduce all-cause mortality by 33% and a combined outcome of stroke, systemic embolism, and death by 48% [141].

The persistent disparity between actual stroke prevention practices in chronic fibrillation patients and published stroke prevention guidelines was recently examined. The results indicated, unfortunately, that anticoagulation therapy aimed to prevent stroke after cardiac or other systemic illness that present high risk of embolism is underused [142]. On the other hand, based on the encouraging results obtained using aspirin, new trials using both aspirin and clopidogrel are being tested at present. Those trails also aim at overcoming aspirin resistance, a growing complication of aspirin treatment. (e.g., reduce the low response incidence of aspirin and clopidogrel therapy after coronary stent). To achieve this aim, 504 patients were included in the study. The antiplatelet therapy included a loading dose of 600 mg clopidogrel and 500 mg aspirin, followed by 75 mg clopidogrel and 100 mg aspirin once daily. The results for clopidogrel revealed that in order to overcome low response to clopidogrel, it would be effective to increase the dose of clopidogrel 150 mg daily. The importance of this study resides in the fact that many laboratory values like elevated C-reactive protein as well as acute coronary syndrome, diabetes mellitus, renal failure, and reduced left ventricular function are risk factors to determine clopidogrel resistance. On the other hand, patients with elevated hemoglobin, serum creatinine, and C-reactive values are directly related to aspirin resistance. So, following a structured therapy plan based on a “test and treat” strategy could significantly reduce the risk of inadequate antiplatelet therapy [143].

## 10. Conclusion

Platelets participate in both hemostasis and thrombosis by forming aggregates on an injured intimal surface. Thus, many platelet receptors and enzymes have been targeted by “antiplatelet” agents, for example, COX-1, which is targeted by aspirin, for therapeutic purposes. Also, recent studies indicate that inhibition of COX-1 is associated with a significant decrease in collagen-induced aggregation of human platelets and to a decreased release of platelet ADP. This characteristic has made aspirin one of the drugs used for prevention of fatal events that could arise as a consequence of thrombotic events. Nevertheless, recent reports have shown an increased number of patients under treatment with aspirin that present with resistance. Consequently, the lack of an effective method of measurement of the levels of antiaggregant effect induced by the chronic use of aspirin in the past has been a limitation for its use for prevention in patients with episodes of transient ischemic disorders or stroke. New antiplatelet drugs, such as the adenosine diphosphate receptor inhibitors or GPIIb/IIIa inhibitors have been developed with the aim to replace aspirin as antiaggregant agent. However, none of these are expected to replace the conventional drugs in multiple therapeutic approaches. ADP receptor inhibitors are used together with aspirin to overcome possible patient resistance, and new protocols are being proposed to replace the older protocols based on trials performed in the last decade. The use of new techniques for determining the levels of antiaggregation obtained after aspirin treatment, like platelets impedance aggregometry, is making possible monitoring the risk of the patient under preventive treatment for stroke. This gives therapists the tools for the adequate use (dose and timing) of aspirin for antiplatelet therapy, by offering more protection against thrombiembolism with lower bleeding risk and simpler dosing and monitoring. New protocols based on those premises are being generated in order to give to the therapist and the patient the level of confidence necessary for a better prevention and treatment of thrombotic events.
